# Redetermination of 4-nitro­stilbene

**DOI:** 10.1107/S1600536808035459

**Published:** 2008-11-08

**Authors:** Rodolfo Moreno-Fuquen, Lina Aguirre, Alan R. Kennedy

**Affiliations:** a Departamento de Química, Facultad de Ciencias, Universidad del Valle, Apartado 25360, Santiago de Cali, Colombia; b Department of Pure and Applied Chemistry, University of Strathclyde, 295 Cathedral Street, Glasgow G1 1XL, Scotland

## Abstract

In the title compound, C_14_H_11_NO_2_, the benzene rings are inclined to each other with a dihedral angle between their mean planes of 8.42 (6)°. The nitro group is almost coplanar with the attached benzene ring but is rotated about the C—N bond by 5.84 (12)°. This redetermination results in a crystal structure with significantly higher precision than the original determination [Hertel & Romer (1931 ▶). *Z. Kristallogr.* **76**, 467–469], and the intermolecular interactions have been established. In the crystal structure, mol­ecules are linked by C—H⋯O hydrogen bonds to generate *C*(5), *C*(13) and edge-fused *R*_3_^3^(28) rings.

## Related literature

For a previous study of the title compound, see: Hertel & Romer (1931 ▶). For background information on photonic materials, see: Luo *et al.* (2003 ▶); Vidal *et al.* (2008 ▶); Park *et al.* (2004 ▶). For general background, see: Allen *et al.* (1987 ▶); Etter (1990 ▶); Nardelli (1995 ▶). 
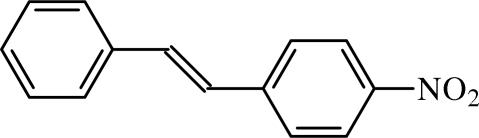


## Experimental

### 

#### Crystal data

C_14_H_11_NO_2_
*M* *_r_* = 225.24
Orthorhombic, 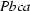

*a* = 10.0839 (3) Å
*b* = 7.6849 (2) Å
*c* = 28.1176 (8) Å
*V* = 2178.94 (11) Å^3^
*Z* = 8
Mo *K*α radiation
μ = 0.09 mm^−1^
*T* = 123 (2) K
0.40 × 0.40 × 0.18 mm

#### Data collection

Oxford Xcalibur-S diffractometer
Absorption correction: multi-scan (*CrysAlis RED*; Oxford Diffraction, 2008 ▶) *T*_min_ = 0.965, *T*_max_ = 0.985
13263 measured reflections
3173 independent reflections
2202 reflections with *I* > 2σ(*I*)
*R*_int_ = 0.027

#### Refinement

*R*[*F* ^2^ > 2σ(*F* ^2^)] = 0.048
*wR*(*F* ^2^) = 0.135
*S* = 1.09
3173 reflections
154 parameters
H-atom parameters constrained
Δρ_max_ = 0.32 e Å^−3^
Δρ_min_ = −0.18 e Å^−3^

### 

Data collection: *CrysAlis CCD* (Oxford Diffraction, 2008 ▶); cell refinement: *CrysAlis RED* (Oxford Diffraction, 2008 ▶); data reduction: *CrysAlis RED*; program(s) used to solve structure: *SIR97* (Altomare *et al.*, 1999 ▶); program(s) used to refine structure: *SHELXL97* (Sheldrick, 2008 ▶); molecular graphics: *ORTEP-3 for Windows* (Farrugia, 1997 ▶); software used to prepare material for publication: *PARST95* (Nardelli, 1995 ▶).

## Supplementary Material

Crystal structure: contains datablocks I, global. DOI: 10.1107/S1600536808035459/hg2430sup1.cif

Structure factors: contains datablocks I. DOI: 10.1107/S1600536808035459/hg2430Isup2.hkl

Additional supplementary materials:  crystallographic information; 3D view; checkCIF report

## Figures and Tables

**Table 1:** Hydrogen-bond geometry (Å, °)

*D*—H⋯*A*	*D*—H	H⋯*A*	*D*⋯*A*	*D*—H⋯*A*
C2—H2⋯O1^i^	0.95	2.55	3.3762 (17)	146
C12—H12⋯O1^ii^	0.95	2.66	3.4139 (16)	137
C12—H12⋯O2^iii^	0.95	2.74	3.4046 (17)	128
C11—H11⋯O2^iii^	0.95	2.90	3.4820 (17)	121

Symmetry codes: (i) 
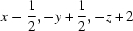
; (ii) 
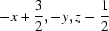
; (iii) 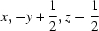.

**Fig. 1.**
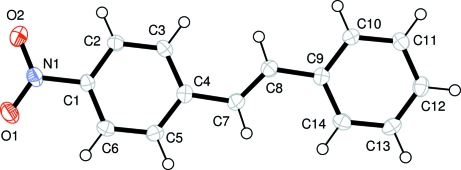
An ORTEP-3 (Farrugia, 1997) plot of the (I) compound, with the atomic labelling scheme. The shapes of the ellipsoids correspond to 50% probability contours of atomic displacement and, for the sake of clarity, H atoms are shown as spheres of arbitrary radius.

**Fig. 2.**
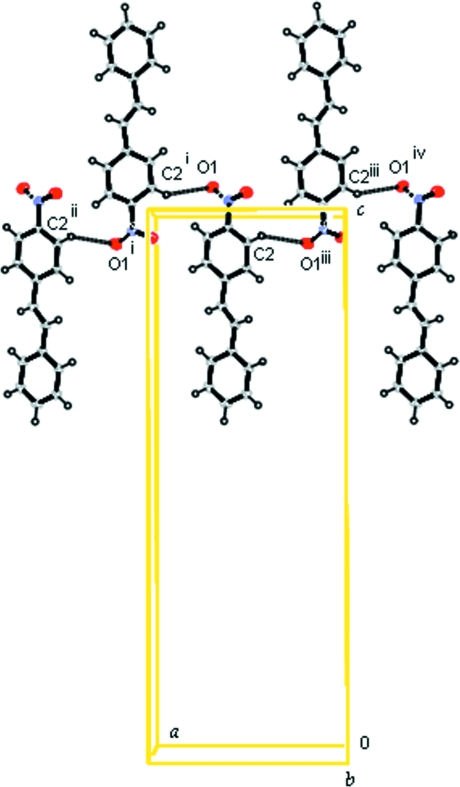
(Part of the crystal structure of (I) showing the formation of C(5) chains which are running parallel to the [100] direction. [Symmetry codes: (i) 1/2 + x, 1/2 - y, 2 - z; (ii) 1 + x, y, z; (iii) -1/2 + x, 1/2 - y, 2 - z; (iv) -1 + x, y, z].

**Fig. 3.**
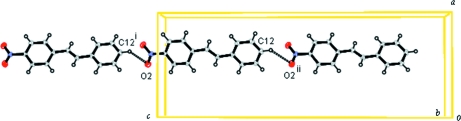
Part of the crystal structure of (I) showing the formation of C(13) chains along [001]. [Symmetry codes: (i) x, 1/2 - y, 1/2 + z; (ii) x, 1/2 - y, -1/2 + z;

**Fig. 4.**
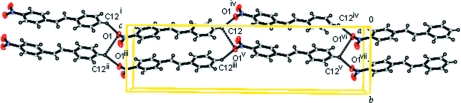
Part of the crystal structure of (I) showing the formation of edge-fused R33(28) rings along [001]. [Symmetry codes: (i) 3/2 - x, -y, 1/2 + z; (ii) x, 1/2 - y, 1/2 + z; (iii) 3/2 - x, 1/2 + y, z; (iv) 3/2 - x, -y, -1/2 + z]; (v) x, 1/2 - y, -1/2 + z; (vi) x, y, -1 + z; (vii) 3/2 - x, y, -1 + z.

## supplementary crystallographic information

### Comment

A great interest in the design of materials with potential applications in photonic technology has been developed in recent years (Luo *et al.*, 2003; Vidal *et al.*, 2008). Significant efforts have been focused on studying of design and the synthesis of organic molecules with potential nonlinear optical response (NLO), improved optical transparency and thermal stability (Park *et al.*, 2004). A specific type of these molecules consists of electron donor and acceptor end groups interacting through a conjugating segment. In a first stage of work in our group, the synthesis of a stilbene molecule with nitro group with electron-withdrawing capacity as a substituent in *para* position, is considered. In order to obtain detailed structural information on the molecular conformation, its NLO responses, its hydrogen bonded interactions and its supramolecular arrangement, the crystal structure of *p*-nitrostilbene (I) was undertaken.

Perspective view of the title molecule, showing the atomic numbering scheme, is given in Fig. 1. The benzene rings are twisted out of the ethylene plane, as defined by the torsion angles C3—C4—C7—C8 and C7—C8—C9—C14 therefore the benzene rings are inclined to each other showing a dihedral angle between their mean planes of 8.42 (6)°. The nitro group is almost coplanar with the benzene ring but it is rotated about the C—N bond with an angle of rotation of 5.84 (12)°. If compared with the C7—C8 bond length to the expected value for a localized double bond [1.317 (13) Å, Allen *et al.*, 1987], the title distance shows some lengthening that is indicative of some π conjugation of the two benzene rings through the central ethene bridge. The torsion angle between the benzene rings [C4—C7═C8—C9 = 178.48 (12)°] indicates a *trans* geometry between them. The crystal structure of (I) is stabilized by weak C—H···O hydrogen-bonding interactions [Nardelli, 1995, Table 1]. The formation of the framework can be explained in terms of three-one substructures. In the first substructure atom C2 in the molecule at (*x*, *y*, *z*) acts as a hydrogen bond donor to nitro atom O1 in the molecule at (-1/2 + *x*, 1/2 - *y*, 2 - *z*) so generating, by 2_1_ screw axes, C(5) chains which are running along [100] (Fig. 2). In the second substructure, atom C12 in the molecule at (*x*, *y*, *z*) acts as hydrogen bond donor to nitro atom O2 in the molecule at (*x*, 1/2 - *y*, -1/2 + *z*) so generating C(13) chains along [001] (Fig. 3). In the third-one dimensional substructure atom C12 in the molecule at (*x*, *y*, *z*) acts simultaneously as hydrogen bond donor to atoms O1 in the molecule at (*x*, 1/2 - *y*, -1/2 + *z*) and atom O1 in the molecule at (3/2 - *x*, -*y*, -1/2 + *z*) so generating a chain of edge-fused with graph motif *R*_3_^3^(28) rings along [001] (Etter, 1990), [Fig. 4].

### Experimental

The synthesis of (I) was prepared by taking equimolar quantities of benzyltriphenylphosphonium bromide (0.9600 g, 2.20 mmol) and 4-nitrobenzaldehyde (0.3355 g, 2.20 mmol). The mixture was stirred and it was taken to reflux in dry THF in a nitrogen atmosphere at 273 K. 3.3 mmol of potassium tert-butoxide was dissolved in 5 ml of t-butanol and this solution was added drop to drop to the phosphonium mixture obtaining a change in the color of the reaction mixture and completion of the reaction after two hours. Single crystals suitable for X-ray analysis were obtained by evaporation at room temperature using ethyl acetate as solvent.

### Refinement

The space group Pbca for *p*-nitrostilbene was assigned from the systematic absences. All H-atoms were located from difference maps and then treated as riding atoms [C—H = 0.93 Å and *U*_iso_(H) = 1.2*U*_eq_(C)].

### Crystal data

**Table:**

C_14_H_11_NO_2_	*D*_x_ = 1.373 Mg m^−^^3^
*M**_r_* = 225.24	Melting point: 421(1) K
Orthorhombic, *P**b**c**a*	Mo *K*α radiation λ = 0.71073 Å
Hall symbol: -P 2ac 2ab	Cell parameters from 5152 reflections
*a* = 10.0839 (3) Å	θ = 2.7–30.7º
*b* = 7.6849 (2) Å	µ = 0.09 mm^−^^1^
*c* = 28.1176 (8) Å	*T* = 123 (2) K
*V* = 2178.94 (11) Å^3^	Cut lathe, light yellow
*Z* = 8	0.40 × 0.40 × 0.18 mm
*F*_000_ = 944	

### Data collection

**Table:**

Oxford Xcalibur-S diffractometer	2202 reflections with *I* > 2σ(*I*)
Radiation source: fine-focus sealed tube	*R*_int_ = 0.027
Monochromator: graphite	θ_max_ = 30.0º
ω scans	θ_min_ = 2.9º
Absorption correction: multi-scan(CrysAlis RED; Oxford Diffraction, 2008)	*h* = −14→12
*T*_min_ = 0.965, *T*_max_ = 0.985	*k* = −10→10
13263 measured reflections	*l* = −37→39
3173 independent reflections	

### Refinement

**Table:**

Refinement on *F*^2^	Secondary atom site location: difference Fourier map
Least-squares matrix: full	Hydrogen site location: inferred from neighbouring sites
*R*[*F*^2^ > 2σ(*F*^2^)] = 0.048	H-atom parameters constrained
*wR*(*F*^2^) = 0.135	*w* = 1/[σ^2^(*F*_o_^2^) + (0.0501*P*)^2^ + 0.491*P*] where *P* = (*F*_o_^2^ + 2*F*_c_^2^)/3
*S* = 1.09	(Δ/σ)_max_ < 0.001
3173 reflections	Δρ_max_ = 0.32 e Å^−^^3^
154 parameters	Δρ_min_ = −0.18 e Å^−^^3^
Primary atom site location: structure-invariant direct methods	Extinction correction: none

### Special details

**Table:**

Experimental. Empirical absorption correction using spherical harmonics, implemented in SCALE3 ABSPACK scaling algorithm.
Geometry. All e.s.d.'s (except the e.s.d. in the dihedral angle between two l.s. planes) are estimated using the full covariance matrix. The cell e.s.d.'s are taken into account individually in the estimation of e.s.d.'s in distances, angles and torsion angles; correlations between e.s.d.'s in cell parameters are only used when they are defined by crystal symmetry. An approximate (isotropic) treatment of cell e.s.d.'s is used for estimating e.s.d.'s involving l.s. planes.

### Fractional atomic coordinates and isotropic or equivalent isotropic displacement parameters (Å^2^)

**Table:**

	*x*	*y*	*z*	*U*_iso_*/*U*_eq_	
O1	0.69853 (10)	0.14689 (14)	1.05469 (3)	0.0360 (3)	
O2	0.51679 (11)	0.28695 (16)	1.04197 (4)	0.0453 (3)	
N1	0.60967 (11)	0.19752 (15)	1.02843 (4)	0.0267 (3)	
C1	0.61582 (12)	0.15108 (16)	0.97771 (4)	0.0209 (3)	
C2	0.51199 (13)	0.19912 (17)	0.94826 (5)	0.0232 (3)	
H2	0.4364	0.2572	0.9607	0.028*	
C3	0.52059 (13)	0.16074 (16)	0.90021 (5)	0.0239 (3)	
H3	0.4496	0.1910	0.8796	0.029*	
C4	0.63344 (13)	0.07749 (15)	0.88166 (4)	0.0227 (3)	
C5	0.73329 (13)	0.02692 (17)	0.91315 (5)	0.0247 (3)	
H5	0.8083	−0.0340	0.9013	0.030*	
C6	0.72573 (13)	0.06320 (16)	0.96111 (5)	0.0244 (3)	
H6	0.7945	0.0285	0.9822	0.029*	
C7	0.65267 (13)	0.04480 (16)	0.83078 (5)	0.0245 (3)	
H7	0.7231	−0.0309	0.8221	0.029*	
C8	0.58014 (13)	0.11181 (16)	0.79561 (4)	0.0236 (3)	
H8	0.5084	0.1851	0.8044	0.028*	
C9	0.60077 (12)	0.08280 (15)	0.74429 (4)	0.0218 (3)	
C10	0.50863 (13)	0.15248 (16)	0.71267 (4)	0.0229 (3)	
H10	0.4342	0.2142	0.7247	0.027*	
C11	0.52360 (13)	0.13330 (17)	0.66384 (5)	0.0253 (3)	
H11	0.4599	0.1819	0.6428	0.030*	
C12	0.63151 (13)	0.04317 (16)	0.64595 (5)	0.0261 (3)	
H12	0.6422	0.0302	0.6126	0.031*	
C13	0.72366 (13)	−0.02793 (17)	0.67678 (5)	0.0263 (3)	
H13	0.7973	−0.0904	0.6645	0.032*	
C14	0.70911 (13)	−0.00852 (16)	0.72570 (5)	0.0246 (3)	
H14	0.7730	−0.0575	0.7466	0.030*	

### Atomic displacement parameters (Å^2^)

**Table:**

	*U*^11^	*U*^22^	*U*^33^	*U*^12^	*U*^13^	*U*^23^
O1	0.0325 (6)	0.0503 (6)	0.0254 (5)	−0.0005 (5)	−0.0072 (5)	0.0044 (5)
O2	0.0382 (6)	0.0687 (8)	0.0291 (5)	0.0153 (6)	−0.0009 (5)	−0.0153 (5)
N1	0.0250 (6)	0.0335 (6)	0.0217 (5)	−0.0050 (5)	−0.0026 (5)	0.0008 (5)
C1	0.0213 (6)	0.0227 (6)	0.0188 (6)	−0.0036 (5)	−0.0003 (5)	0.0011 (5)
C2	0.0191 (6)	0.0269 (6)	0.0236 (6)	0.0000 (5)	−0.0005 (5)	−0.0002 (5)
C3	0.0225 (6)	0.0264 (6)	0.0226 (6)	−0.0018 (5)	−0.0041 (5)	0.0024 (5)
C4	0.0263 (6)	0.0196 (5)	0.0220 (6)	−0.0040 (5)	0.0010 (5)	0.0004 (5)
C5	0.0232 (6)	0.0242 (6)	0.0267 (7)	0.0028 (5)	0.0023 (5)	0.0001 (5)
C6	0.0231 (6)	0.0231 (6)	0.0271 (7)	0.0013 (5)	−0.0022 (5)	0.0039 (5)
C7	0.0244 (6)	0.0237 (6)	0.0253 (6)	0.0008 (5)	0.0014 (5)	−0.0023 (5)
C8	0.0243 (6)	0.0223 (6)	0.0242 (6)	−0.0003 (5)	0.0012 (5)	−0.0004 (5)
C9	0.0254 (6)	0.0177 (5)	0.0222 (6)	−0.0030 (5)	0.0010 (5)	−0.0002 (5)
C10	0.0222 (6)	0.0218 (6)	0.0247 (6)	0.0007 (5)	0.0017 (5)	−0.0005 (5)
C11	0.0282 (7)	0.0238 (6)	0.0240 (6)	−0.0002 (5)	−0.0028 (5)	0.0013 (5)
C12	0.0343 (7)	0.0225 (6)	0.0215 (6)	−0.0026 (6)	0.0027 (6)	−0.0023 (5)
C13	0.0257 (7)	0.0222 (6)	0.0309 (7)	0.0017 (5)	0.0057 (6)	−0.0039 (5)
C14	0.0240 (7)	0.0213 (6)	0.0285 (7)	0.0000 (5)	−0.0038 (5)	0.0012 (5)

### Geometric parameters (Å, °)

**Table:**

O1—N1	1.2246 (14)	C7—H7	0.9500
O2—N1	1.2225 (15)	C8—C9	1.4750 (17)
N1—C1	1.4712 (16)	C8—H8	0.9500
C1—C6	1.3793 (17)	C9—C10	1.3931 (17)
C1—C2	1.3851 (18)	C9—C14	1.3998 (17)
C2—C3	1.3855 (17)	C10—C11	1.3890 (18)
C2—H2	0.9500	C10—H10	0.9500
C3—C4	1.4058 (18)	C11—C12	1.3845 (18)
C3—H3	0.9500	C11—H11	0.9500
C4—C5	1.3960 (18)	C12—C13	1.3833 (19)
C4—C7	1.4653 (17)	C12—H12	0.9500
C5—C6	1.3789 (18)	C13—C14	1.3914 (18)
C5—H5	0.9500	C13—H13	0.9500
C6—H6	0.9500	C14—H14	0.9500
C7—C8	1.3334 (18)		
			
O2—N1—O1	123.46 (11)	C4—C7—H7	117.1
O2—N1—C1	118.07 (11)	C7—C8—C9	126.15 (12)
O1—N1—C1	118.46 (12)	C7—C8—H8	116.9
C6—C1—C2	122.37 (12)	C9—C8—H8	116.9
C6—C1—N1	118.74 (11)	C10—C9—C14	118.36 (12)
C2—C1—N1	118.88 (11)	C10—C9—C8	118.19 (11)
C1—C2—C3	118.62 (12)	C14—C9—C8	123.45 (12)
C1—C2—H2	120.7	C11—C10—C9	121.17 (12)
C3—C2—H2	120.7	C11—C10—H10	119.4
C2—C3—C4	120.62 (12)	C9—C10—H10	119.4
C2—C3—H3	119.7	C12—C11—C10	119.85 (12)
C4—C3—H3	119.7	C12—C11—H11	120.1
C5—C4—C3	118.37 (11)	C10—C11—H11	120.1
C5—C4—C7	118.43 (12)	C13—C12—C11	119.86 (12)
C3—C4—C7	123.18 (11)	C13—C12—H12	120.1
C6—C5—C4	121.61 (12)	C11—C12—H12	120.1
C6—C5—H5	119.2	C12—C13—C14	120.42 (12)
C4—C5—H5	119.2	C12—C13—H13	119.8
C5—C6—C1	118.32 (12)	C14—C13—H13	119.8
C5—C6—H6	120.8	C13—C14—C9	120.34 (11)
C1—C6—H6	120.8	C13—C14—H14	119.8
C8—C7—C4	125.82 (12)	C9—C14—H14	119.8
C8—C7—H7	117.1		
			
O2—N1—C1—C6	174.44 (12)	C5—C4—C7—C8	−167.03 (12)
O1—N1—C1—C6	−4.53 (17)	C3—C4—C7—C8	11.53 (19)
O2—N1—C1—C2	−4.71 (18)	C4—C7—C8—C9	178.48 (12)
O1—N1—C1—C2	176.32 (11)	C7—C8—C9—C10	174.99 (12)
C6—C1—C2—C3	−1.49 (19)	C7—C8—C9—C14	−6.1 (2)
N1—C1—C2—C3	177.63 (11)	C14—C9—C10—C11	−0.39 (18)
C1—C2—C3—C4	−1.10 (18)	C8—C9—C10—C11	178.59 (11)
C2—C3—C4—C5	3.13 (18)	C9—C10—C11—C12	0.17 (18)
C2—C3—C4—C7	−175.43 (12)	C10—C11—C12—C13	0.24 (19)
C3—C4—C5—C6	−2.73 (18)	C11—C12—C13—C14	−0.42 (19)
C7—C4—C5—C6	175.91 (11)	C12—C13—C14—C9	0.19 (18)
C4—C5—C6—C1	0.27 (19)	C10—C9—C14—C13	0.21 (17)
C2—C1—C6—C5	1.90 (19)	C8—C9—C14—C13	−178.71 (11)
N1—C1—C6—C5	−177.22 (11)		

### Hydrogen-bond geometry (Å, °)

**Table:**

*D*—H···*A*	*D*—H	H···*A*	*D*···*A*	*D*—H···*A*
C2—H2···O1^i^	0.95	2.55	3.3762 (17)	146
C12—H12···O1^ii^	0.95	2.66	3.4139 (16)	137
C12—H12···O2^iii^	0.95	2.74	3.4046 (17)	128
C11—H11···O2^iii^	0.95	2.90	3.4820 (17)	121

Symmetry codes: (i) *x*−1/2, −*y*+1/2, −*z*+2; (ii) −*x*+3/2, −*y*, *z*−1/2; (iii) *x*, −*y*+1/2, *z*−1/2.

## References

[bb1] Allen, F. H. (2002). *Acta Cryst.* B**58**, 380–388. 10.1107/s0108768102003890 12037359

[bb2] Allen, F. H., Kennard, O., Watson, D. G., Brammer, L., Orpen, A. G. & Taylor, R. (1987). *J. Chem. Soc. Perkin Trans. 2*, pp. S1–19.

[bb3] Altomare, A., Burla, M. C., Camalli, M., Cascarano, G. L., Giacovazzo, C., Guagliardi, A., Moliterni, A. G. G., Polidori, G. & Spagna, R. (1999). *J. Appl. Cryst.* **32**, 115–119.

[bb4] Etter, M. (1990). *Acc. Chem. Res.* **23**, 120–126.

[bb5] Farrugia, L. J. (1997). *J. Appl. Cryst.* **30**, 565.

[bb6] Hertel, E. & Romer, G. H. (1931). *Z. Kristallogr.* **76**, 467–469.

[bb7] Luo, J., Haller, M., Li, H., Kim, T.-D. & Jen, A. K.-Y. (2003). *Adv. Mater.* **15**, 1635–1638.

[bb8] Nardelli, M. (1995). *J. Appl. Cryst.* **28**, 659.

[bb9] Oxford Diffraction (2008). *CrysAlis CCD* and *CrysAlis RED* Oxford Diffraction, Wrocław, Poland.

[bb10] Park, G., Jung, W. S. & Ra, C. S. (2004). *Bull. Korean Chem. Soc.* **25**, 1427–1429.

[bb11] Sheldrick, G. M. (2008). *Acta Cryst.* A**64**, 112–122. 10.1107/S0108767307043930 18156677

[bb12] Vidal, X., Fedyanin, A., Molinos-Gomez, A., Rao, S., Martorell, J. & Petrov, D. (2008). *Opt. Lett.* **33**, 699–701. 10.1364/ol.33.000699 18382522

